# Lateral prefrontal model-based signatures are reduced in healthy individuals with high trait impulsivity

**DOI:** 10.1038/tp.2015.139

**Published:** 2015-10-13

**Authors:** L Deserno, T Wilbertz, A Reiter, A Horstmann, J Neumann, A Villringer, H-J Heinze, F Schlagenhauf

**Affiliations:** 1 Max Planck Fellow Group ‘Cognitive and Affective Control of Behavioral Adaptation', Max Planck Institute for Human Cognitive and Brain Sciences, Leipzig, Germany; 2 Department of Psychiatry and Psychotherapy, Campus Charité Mitte, Charité—Universitätsmedizin Berlin, Berlin, Germany; 3 Department of Neurology, Otto-von-Guericke University, Magdeburg, Germany; 4 International Max Planck Research School on the Neuroscience of Communication, Leipzig, Germany; 5 Department of Cognitive Neurology, Max Planck Institute for Human Cognitive and Brain Sciences, Leipzig, Germany; 6 IFB AdiposityDiseases, University of Leipzig, Leipzig, Germany; 7 Clinic for Cognitive Neurology, University Hospital Leipzig, Leipzig, Germany; 8 Berlin School of Mind and Brain, Mind and Brain Institute, Humboldt University, Berlin, Germany; 9 Department of Behavioral Neurology, Leibniz Institute for Neurobiology, Magdeburg, Germany

## Abstract

High impulsivity is an important risk factor for addiction with evidence from endophenotype studies. In addiction, behavioral control is shifted toward the habitual end. Habitual control can be described by retrospective updating of reward expectations in ‘model-free' temporal-difference algorithms. Goal-directed control relies on the prospective consideration of actions and their outcomes, which can be captured by forward-planning ‘model-based' algorithms. So far, no studies have examined behavioral and neural signatures of model-free and model-based control in healthy high-impulsive individuals. Fifty healthy participants were drawn from the upper and lower ends of 452 individuals, completing the Barratt Impulsiveness Scale. All participants performed a sequential decision-making task during functional magnetic resonance imaging (fMRI) and underwent structural MRI. Behavioral and fMRI data were analyzed by means of computational algorithms reflecting model-free and model-based control. Both groups did not differ regarding the balance of model-free and model-based control, but high-impulsive individuals showed a subtle but significant accentuation of model-free control alone. Right lateral prefrontal model-based signatures were reduced in high-impulsive individuals. Effects of smoking, drinking, general cognition or gray matter density did not account for the findings. Irrespectively of impulsivity, gray matter density in the left dorsolateral prefrontal cortex was positively associated with model-based control. The present study supports the idea that high levels of impulsivity are accompanied by behavioral and neural signatures in favor of model-free behavioral control. Behavioral results in healthy high-impulsive individuals were qualitatively different to findings in patients with the same task. The predictive relevance of these results remains an important target for future longitudinal studies.

## Introduction

Impulsivity can be defined as a tendency for premature choices without foresight but despite adverse consequences.^1, 2^ Impulsivity, a multifaceted construct, has been established as a vulnerability factor for addiction.^3^ Recent studies support the view of self-reported trait impulsivity as an endophenotype for addiction disorders:^4, 5^ non-addicted, albeit cognitively impaired and at-risk, first-degree family members showed intermediate levels of trait impulsivity when compared with addicted siblings and unrelated controls.^4, 5^ This endophenotype research characterized unaffected siblings by intermediate brain alterations, most prominently by means of structural measures of frontostriatal circuits.^6^ Interestingly, frontostriatal structural measures were shown to correlate with the expression of the dominant mode of behavioral control.^7, 8^ An important proposal linked the personality trait impulsivity to an overreliance on habitual behavioral control.^9, 10^ Empirical evidence for this hypothesis mainly stems from animal models of drug addiction showing that high-impulsive rats are predisposed for escalation of repeated drug self-administration and early relapse after abstinence.^11^

Behavioral control is postulated to be parsed between competing habitual and goal-directed systems.^12, 13^ This dual system theory was formalized through the use of computational models:^14^ habitual control can be described by ‘model-free' temporal-difference algorithms, which retrospectively update expectations by reward prediction errors. Dominance of model-free control is accompanied by reduced immediate sensitivity to outcome devaluation because new outcome experiences are required to gradually adapt outcome expectations.^15^ In marked contrast, goal-directed control relies on the prospective consideration of possible actions and their potential future outcomes.^16^ This can be described by ‘model-based' algorithms, which capture a task as a map in a forward-planning manner and therefore model-based control enables flexible behavioral adaptation in dynamic environments.^17^ Using sequential decision-making and computational modeling, it was demonstrated that healthy individuals use a mixture of both control strategies, meanwhile prefrontal cortex (PFC) and ventral striatum code signatures of both model-free and also model-based control.^18, 19^ Strikingly, when using the same task, a balance of behavioral control shifted toward model-free control was reported across several psychiatric conditions characterized by high levels of trait impulsivity, including addiction.^7, 20^

Adopting such a *Computational Psychiatry* approach,^21, 22, 23^ it has yet not been studied whether a shift toward model-free control also extends to the vulnerability factor impulsivity. One study could show that high-impulsive smokers showed reduced goal-directed control in a devaluation paradigm when compared with low-impulsive smokers.^24^ However, the latter study could not rule out potential effects of smoking addiction and did not include functional or structural brain measures. To fill this gap, we utilized sequential decision-making, as in previous studies,^7, 18, 19, 20^ in healthy low- and high-impulsive individuals taken from a larger sample. Finally, 50 participants underwent task-based functional magnetic resonance imaging (fMRI) to examine neural correlates of model-free and model-based control based on computational modeling of the observed behavior. First, we explored whether high-impulsive individuals show reduced model-based control similar to patients.^7, 20^ Dimensional approaches to psychiatry suggest that impairments in behavioral control, as observed in drug addiction, could lie at the end of a continuum including healthy high-impulsive individuals.^2^ Therefore, it appears conceivable that healthy individuals with levels of impulsivity comparable to patients show intermediate alterations in behavioral control. Second, on the neural level, we tested whether high-impulsive individuals show elevated model-free prediction errors or reduced model-based signatures. Such effects were expected in ventral striatum or PFC, as these regions were previously indicated in coding model-free prediction errors and additional model-based signatures.^18, 19^ Structural MRI was analyzed by means of gray matter density to assess its covariation with the behavioral and functional imaging effects.

## Materials and methods

### Participants and instruments

A total of 452 participants completed the Barratt Impulsiveness Scale (BIS)-11, a self-reported measurement of trait impulsivity with high retest reliability in clinical and non-clinical populations.^25^ Among these, 52 right-handed individuals were selected from the upper and lower ends. Sample size for this study was determined in accordance to previous between-group studies with the same task.^7, 20^ According to the literature,^26^ the mean total BIS scores of each group met criteria for high and low impulsiveness (Table 1). Both groups were matched for age and gender and screened for axis-I psychiatric disorders using the Structured Clinical Interview for DSM Disorders IV (SCID-IV) interview.^27^ On the basis of this screening, one participant was excluded because of a recent episode of major depression and another participant fell asleep during task-based fMRI. The final sample consisted of 50 participants (24 high-impulsive and 26 low-impulsive participants). Intelligence was examined based on a German vocabulary test^28^ as well as working memory using the backward digit span test and processing speed using the digit symbol substitution test.^29^ Drinking was assessed with the time-line follow-back interview.^30^ For detailed group description see Table 1. The local ethics committee (University Leipzig) approved the study. All participants gave written informed consent and received monetary compensation on an hourly basis in addition to their monetary gain during the task. We have not replicated the effects of impulsivity on behavioral and neural signatures in our laboratory.

### Sequential decision-making task

**Figure 1:**
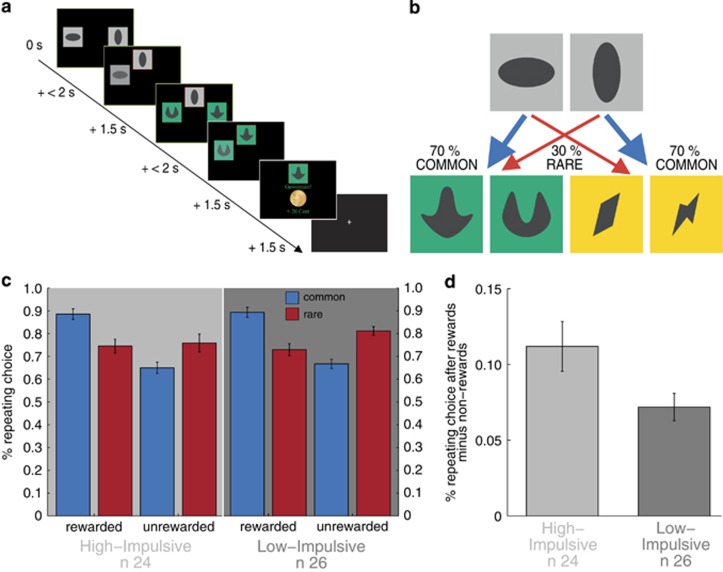
Task and behavioral raw data. (**a**) Exemplary trial sequence of the task. (**b**) State-transition probabilities. (**c**) Stay-switch behavior at the first stage was analyzed as a function of reward and state in the previous trial. These stay probabilities were subjected to repeated-measures analysis of variance (ANOVA) with reward and state as within-subject factors and group as between-subject factors. This revealed a significant main effect of reward (F(1,48)=75.30, *P*<0.001) and reward × state interaction (F(1,48)=64.30, *P*<0.001); no significant main effect of state (F(1,48)=1.32, *P*=0.26) and no significant state × group (F(1,48)=0.07, *P*=0.80) or reward × state × group (F(1,48)=0.73, *P*=0.40) interactions. There was a trend toward a significant reward × group interaction (F(1,48)=3.50, *P*=0.07). (**d**) In a one-tailed *post hoc* *t*-test, the difference between staying after rewards and staying after non-rewards was significantly increased in the high- compared with the low-impulsive group (*T*(48)=1.94, *P*=0.04). Error bars represent s.e.

A two-step sequential decision-making task was implemented as in previous studies.^7, 18, 31^ Participants had to make two sequential choices between pairs of stimuli to receive a monetary reward after the second choice. Within each trial, participants had to decide between two gray boxes at the first stage or two colored boxes at the second stage (Figure 1a). Crucially, each first-stage choice was associated with a different pair of colored boxes at the second stage via a fixed transition probability of 70%, which did not change during the experiment (Figure 1b). Thus, choice of each first-stage stimulus was *commonly* (70%) associated with a certain second-stage pair of stimuli and this is labeled a ‘common state'. In reverse, choice of each first-stage stimulus *rarely* resulted (30%) in the other second-stage pair of stimuli and this is labeled a ‘rare state'. Model-free control neglects this transition probability and staying with the same first-stage action that lead to a reward after a second-stage choice is most likely (a main effect of reward). In contrast, model-based control takes into account the transition probabilities. Thus, staying at the first-stage decreases after having received a reward in a rare state but increases after having received no reward in a rare state (reward × state interaction).

All stimuli were randomly assigned to the left and right positions on the screen. At the first stage, the chosen gray stimulus was surrounded with a red frame, moved to the top of the screen after completion of a 2- s decision phase and remained there for 1.5 s. Subsequently, participants entered the second stage (a common or rare state depending on the type of transition) and decided between two colored boxes. After a second-stage choice, feedback (reward or no reward) was delivered according to slowly and independently changing Gaussian random walks. These random walks were identical to Daw *et al.*,^18^ as it was shown that less distinct random walks for reward delivery reduce the degree of model-based behavior.^32^ Slowly changing reward probabilities at the second stage challenge the subject with ongoing learning and thus maximize the dissociation of the two control strategies at the first stage. Thus, non-stationary reward probabilities at the second stage induce ongoing model-based evaluation, whereas stationary reward probabilities would favor a dominance of model-free control at some point in time. The task consisted of a total of 201 trials with two choice stages within each trial. Trials were separated by an exponentially distributed intertrial interval with a mean of 2 s. Before the experiment and similar to Daw *et al.*, participants were explicitly informed that the transition structure from the first to the second stage would remain constant throughout the task. Information was provided about the independence of reward probabilities and their change over time. Before MRI scanning, participants performed a 55-trial version of the task with different stimuli and reward probabilities and were instructed to maximize reward in the main experiment, which they received as monetary payout after completion of the task.

First-stage stay-switch behavior was analyzed as a function of reward (reward/no reward) and state (common/rare) in the previous trial. Each individual's first-stage stay probabilities were subjected to repeated-measures analysis of variance (ANOVA, using anovan in Matlab) with reward and state as within-subject factors and impulsivity (high/low) as between-subject factors. A main effect of reward shows an influence of model-free control, whereas the interaction of reward and state reveals influences of model-based control. Previously, healthy individuals showed a mixture of both control strategies^7, 18, 19, 20, 31, 33^ expressed by a significant main effect of reward and a significant interaction of reward and state. In the following, we describe a more fine-grained dissociation of the two control strategies via computational modeling, which also provides individual trial-by-trial signatures for the analysis of neural measurements. All behavioral analyses were performed using Matlab 2010b (The MathWorks, Natick, Massachusetts, USA).

### Computational model

As in previous studies,^7, 18, 19, 31^ we adopted a computational modeling approach to disentangle influences of model-free and model-based control on participant's choice behavior. To this end, three types of models were applied. (1) A model-free algorithm capturing only a main effect of reward in first-stage stay-switch behavior. This algorithm was the temporal-difference model SARSA(λ), which learns decision values *retrospectively* after prediction errors occur.^34^ (2) A model-based algorithm, which only gives an interaction of reward and state but no main effect of reward. To this end, first-stage values were computed *prospectively* by multiplying maximum values at the second stage with explicitly instructed transition probabilities.^18^ (3) A combination of both algorithms, a so-called hybrid model that can reproduce a main effect of reward and an interaction of reward and state.^18^ Values from all three models were transformed into choice probabilities using a softmax rule with three parameters accounting for stochasticity separately at the first and second stages (β_1 and 2_) and a repetition parameter (*ρ*) accounting for perseverance of first-stage choices.

Leaving out parameters of the softmax, the model-free algorithm SARSA(λ) has three parameters: first- and second-stage learning rates (*α*_1_/*α*_2_), which describe how quickly values change with respect to first-stage and second-stage prediction errors; stage-skipping update *λ* (another learning rate), which connects the two stages via an influence of reward prediction errors at the second-stage on first-stage values. Importantly, *λ* describes how quickly first-stage values change with respect to second-stage reward prediction errors and thus accounts for the main effect of reward in first-stage stay behavior but not for an interaction of reward and state. Thus, a high value of *λ* signifies a stronger influence of reward prediction errors at the second stage on first-stage values. The model-based algorithm shares one parameter with the model-free algorithm (*α*_2_) because both algorithms converge at the second stage. In line with previous work,^18, 19^ we also show that including the parameter *λ* improves the fit to the data (see Supplementary Table S1). To give an interaction of reward and state no further parameter is required as the interaction results from multiplying maximum values of second-stage stimuli with explicitly instructed transition probabilities.^18^ The hybrid algorithm has a total of four parameters: three parameters from SARSA(*λ*) and a fourth parameter (*ω*) that weights the influence of model-free and model-based values and is therefore of most interest because it represents a relative balance of the two control strategies. Please see Supplementary Information for equations and model fitting.

### Model comparison

The aim of model comparison is to identify one best-fitting algorithm. In other words, a control strategy that is most likely in groups of high- and low-impulsive individuals. To compare the three models for their relative goodness of fit, we subjected the model evidence (approximated via sampling from the empirical prior distribution) to a random-effects Bayesian model selection procedure.^35^ The resulting exceedance probabilities show which model is most likely in a population.^35^ In the Supplementary Information, we show that other measurements of relative model fit proved consistent with this approach (Supplementary Table S1) and show that best-fitting parameters reproduce the observed behavior (Supplementary Figure S1).

### Group comparison of model parameters

The predictions of the two control strategies differ at the first stage of the task. In accordance with raw data analysis, parameters that explain variance in first-stage decision values are of main interest here. In the hybrid model, the winning model in both groups, a weighting parameter (*ω*) determines to which extent overall first-stage decision values are influenced by model-free and model-based values. Two further parameters, originally from the model-free algorithm, also directly influence the update of first-stage values: first-stage learning rate (*α*_1_), which determines how quickly first-stage values change with respect to prediction errors at the onset of the second stage, and a stage-skipping update (*λ*), which determines to what extent first-stage values change with respect to reward prediction errors and accounts for the main effect of reward. Finally, there is also a second-stage learning rate (*α*_2_), which determines how quickly second-stage values change with respect to reward prediction errors but do not directly influence first-stage values; we subjected all four parameters of the hybrid model (*ω*, *α*_1,_ *α*_2,_ *λ*) to a one-way multivariate ANOVA (MANOVA, using manova1 in Matlab) with the between-subject factor impulsivity (high/low).

### Magnetic resonance imaging

Functional imaging was performed using a 3-Tesla Siemens Trio scanner to acquire gradient echo T2*-weighted echo-planar images with blood oxygenation level-dependent contrast. Covering the whole brain, 36 slices were acquired in oblique orientation at 20° to the anterior commisure-posterior comissure line line in ascending order with 2.5-mm thickness, 3x3 mm^2^ in-plane voxel resolution, 0.5-mm gap between slices, repetition time (TR)=2 s, echo time (TE)=22ms and a flip angle *α*=90°. Before functional scanning, a field map was collected to account for individual homogeneity differences of the magnetic field. T1-weighted structural images were also acquired (TR=1300 ms, TE=3.46 ms, flip=10°, matrix=240 × 256, voxel size: 1 × 1 × 1mm and slices=170).

### Analysis of fMRI data

Two participants had to be excluded because of artifacts in ventral sections of the brain. Thus, functional imaging results are reported for a sample of 48 participants (23 high-impulsive and 25 low-impulsive participants). fMRI data were analyzed using SPM8 (http://www.fil.ion.ucl.ac.uk/spm/software/spm8/). For preprocessing, images were corrected for delay of slice time acquisition. Voxel-displacement maps were estimated based on field maps. All images were realigned to correct for motion and were also corrected for distortion and the interaction of distortion and motion. The images were spatially normalized into the Montreal Neurological Institute space using the normalization parameters generated during the segmentation of each subject's anatomical T1 scan;^36^ spatial smoothing was applied with an isotropic Gaussian kernel of 6-mm full width at half maximum.

Before statistical analysis, data were high-pass-filtered with a cutoff of 128 s. An event-related analysis was applied to the images on two levels using the general linear model approach as implemented in SPM8. As in the original paper by Daw *et al.*^18^ the analysis focused on the two time points within each trial when prediction errors arise: at onsets of the second stage and at onsets of reward delivery. Prediction errors at second-stage onsets compare values of first- and second-stage stimuli and therefore vary with respect to the weighting parameter (*ω*), which gives the balance of the two control strategies. At the first level, both time points were entered into the model as one regressor, which was parametrically modulated (1) by model-free prediction errors and (2) by the difference of model-based and model-free prediction errors, which reflects the difference between model-based and model-free values (the partial derivative of the value function with respect to *ω*). Note that this difference regressor equals zero at reward delivery because both algorithms converge at this time point. To avoid any confound of the neural results because of activity differences between these two time points *per se*, the difference regressor was mean-centered within each subject and the time point of reward delivery was additionally included as a separate regressor. As in Daw *et al.*,^18^ the design also included first-stage onsets with two parametric modulators, the softmax probability for choosing one of the two first-stage probabilities as well as its partial derivative with respect to *ω*; however, these onsets were not in the focus of the present analysis. Individual (random effects) model parameters were used to generate modeling-derived regressors. Invalid trials (no choice within response window) were modeled separately. All regressors were convolved with the canonical hemodynamic response function as provided by SPM8 and its temporal derivative. The six movement parameters from the realignment were included in the model as regressors of no interest as well as the first derivative of translational movement with respect to time. An additional regressor was included censoring scan-to-scan movement >1 mm.

At the second level, contrast images of model-free prediction errors and the difference of model-based and model-free prediction errors were taken to a second-level random-effects model. For correction of multiple comparisons, family-wise error (FWE) *P*<0.05 at the cluster level was applied to statistical maps displayed at *P*<0.001 uncorrected with a cluster extent *k*=20. Previous research revealed an important role of PFC and ventral striatum in coding signatures of both systems.^18, 19, 37, 38^ Thus, the mean parameter estimates for clusters of the ventral striatum and PFC were extracted and then tested between groups using three repeated-measures ANOVAs with control mode (model-free/model-based) as the within-subject factor and impulsivity (high/low) as the between-subject factor. Subsequently, a one-way MANOVA with the between-subject factor impulsivity was used to assess regional specificity by comparing the difference between both effects (model-free prediction errors and the difference of model-based and model-free prediction errors) in all three regions of interest.

### Voxel-based morphometry

For segmentation of each subject's anatomical T1, the unified segmentation approach was applied as implemented in SPM8.^36^ Subsequently, each individual's modulated image of gray matter density was smoothed with an isotropic Gaussian kernel of 6-mm full width at half maximum. The smoothed images were then subjected to a random-effects model containing total intracranial volume as a covariate.

Using fMRI clusters named above, gray matter density was extracted for medial and lateral PFC as well as ventral striatum and included as covariates in between-group comparisons of behavioral and functional imaging data. We also tested for between-group effects. Independent of impulsivity, we examined a covariation of the parameter ω with gray matter density as reported previously for medial prefrontal and orbitofrontal cortex.^7^ Given these results^7^ but also studies that implicate lateral PFC in model-based control,^33^ we constructed a bilateral search volume (taken from the AAL Atlas^39^) of medial prefrontal and orbitofrontal cortex (superior medial frontal gyrus, medial orbitofrontal gyrus and anterior cingulate cortex) and lateral PFC (middle frontal gyrus and inferior frontal gyrus).

## Results

### Sample characteristics

As BIS was the selection criterion, groups differed significantly (Table 1). Notably, the mean BIS of high-impulsive individuals (74.76±4.96) lays in a similar range as for drug users and their siblings.^4^ As shown in Table 1, groups were matched for age and gender and did not differ regarding measures of drinking and smoking or neurocognitive measures.

### Behavioral raw data

First-stage choice behavior of all participants showed a significant main effect of reward and an interaction of reward and state (reward F(1,49)=75.30, *P*<0.001, reward × state F(1,49)=64.30, *P*<0.001, Figure 1c) indicating that across all participants aspects of model-free and model-based control were present. These effects were also present when looking at both groups separately.

Individuals with high trait impulsivity did not show a reduction of model-based control tested by a three-way interaction (reward × state × impulsivity F(1,49)=0.73, *P*=0.40, Figure 1c); however, there was a trend toward a significant reward × impulsivity interaction (F(1,49)=3.50, *P*=0.07, Figure 1c). Close inspection of Figure 1c suggest that this reward × impulsivity interaction results from slightly lower stay probabilities in high-impulsive compared with low-impulsive individuals after unrewarded (particularly unrewarded-rare trials) but not rewarded trials. Thus, the main effect of reward appeared slightly stronger in high-impulsive individuals. To confirm this, a one-tailed between-group *t*-test (high>low) was performed on the main effect of reward (the difference between staying after rewards and staying after non-rewards). Indeed, this difference between staying after rewards and staying after non-rewards was significantly higher in high-impulsive individuals (T(48)=1.94, *P*=0.04, Figure 1d), indicating a subtle accentuation of model-free control in high-impulsive individuals. Although the repeated-measures ANOVA did not reveal any interaction of impulsivity with state or reward and state, following a reviewer's suggestion, we further unpacked the reward × impulsivity interaction for rare and common trials separately. This one-tailed *post hoc* test revealed that the observed effect was mainly driven by the difference between rewarded and unrewarded trials in rare transitions T(48)=1.6, *P*=0.06) but not in common transitions (T(48)=0.26, *P*=0.40).

### Computational modeling

Model selection revealed the hybrid model as best-fitting in both groups (high-impulsive exceedance probability=0.9974, low-impulsive exceedance probability=0.9997, Supplementary Table S1). This underlines that a mixture of both control modes, provided by this hybrid model, is the most likely control mechanisms in low- and high-impulsive groups.

**Figure 2:**
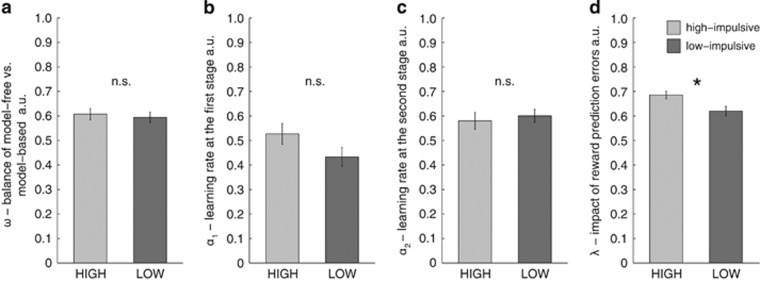
Hybrid model parameters. Four parameters of the hybrid model (*ω*, *α*_1,_ *α*_1_, *λ*, Supplementary Table S2) were subjected to a multivariate analysis with the between-subject factor impulsivity. This revealed a significant effect of impulsivity (F(45)=2.72, *P*=0.04). *Post hoc* univariate tests showed no difference for (**a**) the balance of model-free and model-based control (*ω*), (**b**) for first-stage learning rates (*α*_1_) nor (**c**) for second-stage learning rates (*α*_2_), but (**d**) significantly higher stage-skipping update (*λ*). Error bars represent s.e. *Significant at *P*<0.05. n.s., non-significant.

All four parameters of the hybrid model (*ω*, *α*_1,_ *α*_2,_ *λ*, for their distribution see Supplementary Table S2) were subjected to a MANOVA with the between-subject factor impulsivity. This revealed a significant effect of impulsivity (Δ_Roy_=0.24, F(45)=2.72, *P*=0.041). *Post hoc* univariate tests (Figure 2) showed no difference for the balance of control *ω* (high-impulsive 0.6076±0.1114, low-impulsive 0.5943±0.1080, F(1,48)=0.19, *P*=0.64, Figure 2a), for first-stage learning rates *α*_1_ (high-impulsive 0.5272±0.2077, low-impulsive 0.4330±0.1928, F(1,48)=2.8, *P*=0.10, Figure 2b) nor for second-stage learning rates *α*_2_ (high-impulsive 0.5803±0.1706, low-impulsive 0.6006±0.1328, F(1,48)=0.22, *P*=0.64, Figure 2c), but significantly higher stage-skipping update *λ* (high-impulsive 0.6854±0.0756, low-impulsive 0.6202±0.0965, F(1,48)=7.00, *P*=0.01, Figure 2d). In addition, to demonstrate that the effect of impulsivity on *λ* was not due to fitting *ω* simultaneously, we also tested whether *λ* was significantly different between groups when comparing parameters of the model-free algorithm with *ω*=0. This was indeed the case (high-impulsive 0.69±0.07, low-impulsive 0.64±0.07, T(1,48)=2.96, *P*=0.005). The parameter *λ* signifies a stronger influence of reward prediction errors at the second stage on first-stage decision values and accounts for the main effect of reward observed in first-stage stay behavior. In line with raw data analysis, this speaks for a subtle, albeit significant, elevation of model-free control in high-impulsive individuals. This result remained significant when including neurocognitive measures, amount of alcohol intake or gray matter density as covariates. Explorative comparison of parameters of the softmax observation model (*β*_1_, *β*_2_, *ρ*) and the negative log-likelihood showed no significant differences (T(48)⩽1.61, *P⩾*0.11). See Supplementary Table S2, for distribution of all parameters and the negative log-likelihood.

### Functional MRI

**Figure 3:**
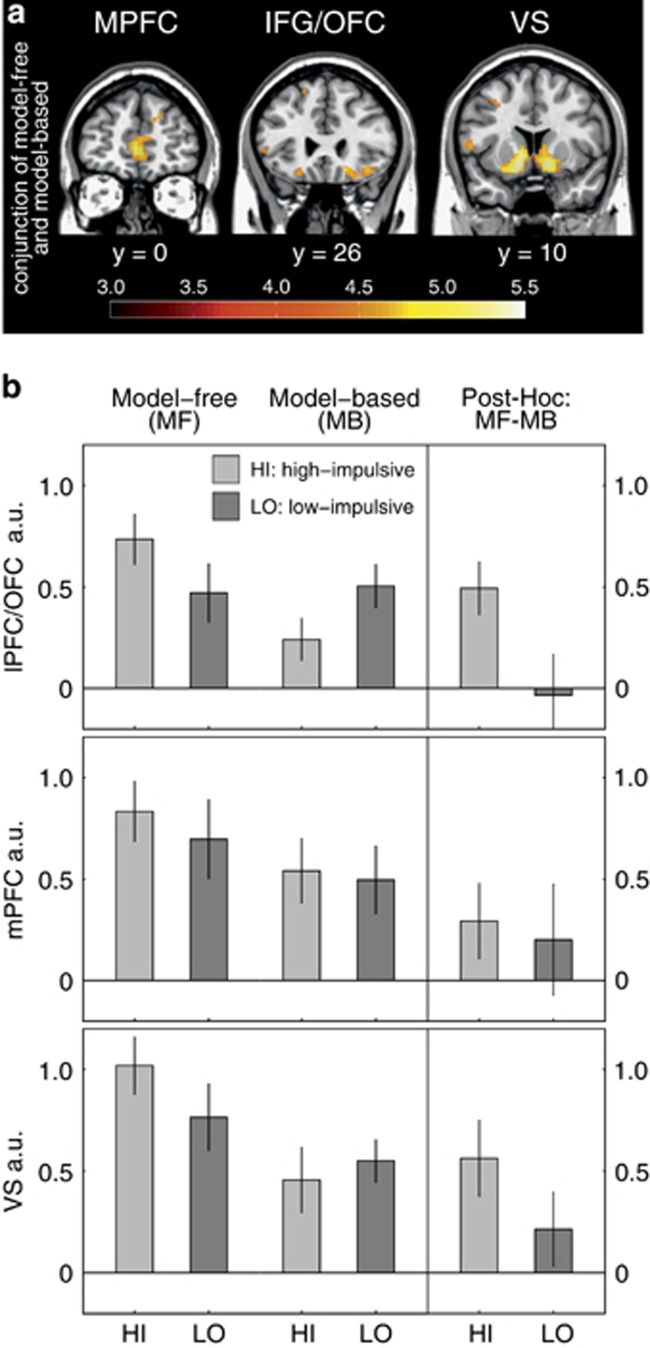
Functional magnetic resonance imaging (fMRI) results across the entire sample. (**a**) Across both groups a significant (whole-brain p-family-wise error (FWE)<0.05 at the cluster level) conjunction of model-free reward prediction errors and the difference of model-based and model-free prediction errors was observed in the right and left ventral striatum (VS), medial prefrontal cortex (mPFC), right ventrolateral prefrontal/orbitofrontal cortex (OFC), right and left parietal cortex and posterior cingulate cortex. For display purposes, maps are thresholded at *P*<0.001 uncorrected and a cluster extent of *k*=20. (**b**) The mean parameter estimates of the cluster for bilateral ventral striatum, medial and right ventrolateral PFC tested between groups using three repeated-measures analysis. No main effect of impulsivity (F(1,46)⩽0.28, *P*⩾0.60) nor an impulsivity × control interaction was observed (F(1,46)⩽1.79, *P*⩾0.19) in the ventral striatum and medial PFC (**b**, middle and lower panel). In right lateral PFC (**b**, upper panel), we observed no main effect of impulsivity (F(1,46)<0.01, *P*=0.99) but a significant impulsivity × learning interaction (F(1,46)=4.80, *P*=0.03). IFG, inferior frontal gyrus; lPFC, lateral prefrontal cortex; n.s., non-significant.

As a replication of previous work,^18, 19^ the conjunction of model-free prediction errors and the difference of model-based and model-free prediction errors across both groups reached significance (whole-brain *P*-FWE<0.05 at the cluster level) in right and left ventral striatum, medial PFC and right ventrolateral prefrontal/orbitofrontal cortex (Supplementary Table S3, Figure 3). Thus, for between-group comparison, parameter estimates of the clusters for the bilateral ventral striatum, medial and right ventrolateral PFC were tested between groups using three repeated-measures ANOVA with control (model-free/model-based) as within-subject factor and impulsivity (high/low) as between-subject factor. As depicted in Figure 3, no main effect of impulsivity (F(1,46)⩽0.28, *P*⩾0.60) nor an impulsivity × control interaction was observed (F(1,46)⩽1.79, *P*⩾0.19) in the ventral striatum and medial PFC. In right lateral PFC, we observed no main effect of impulsivity (F(1,46)<0.01, *P*=0.99, Figure 3) but a significant impulsivity × learning interaction (F(1,46)=4.80, *P*=0.03, Figure 3). To assess regional specificity, MANOVA with the between-subject factor impulsivity was used to compare the difference between two effects of interest (model-free prediction errors and the difference of model-based and model-free prediction errors) in all three regions of interest, which indeed reached significance (Δ_Roy_=0.28, F(44)=4.09, *P*=0.01). All between-group fMRI findings remained significant when adding neurocognitive measures, amount of alcohol intake or gray matter density as covariates.

### Structural MRI

**Figure 4:**
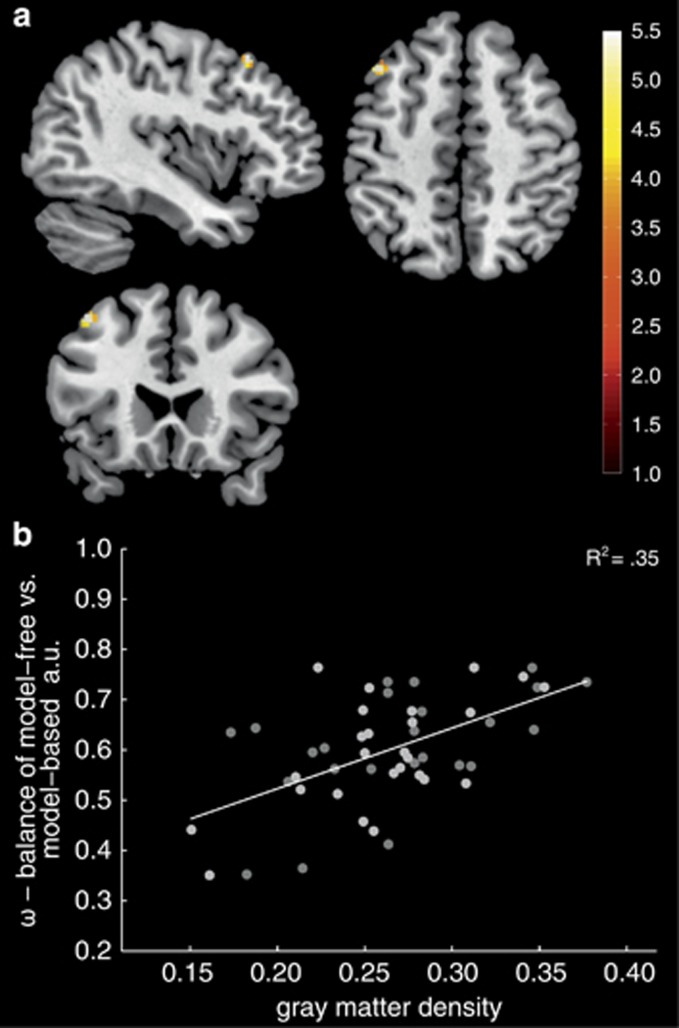
Gray matter density and the balance of behavioral control. (**a**) A positive correlation between gray matter density in the dorsolateral prefrontal cortex (PFC; Montreal Neurological Institute (MNI) *x*=−42, *y*=22, *z*=50, *t*=5.04, p-FWE=0.05 for bilateral medial and lateral PFC) and the balance of model-free and model-based control (*ω*) was observed. For display purposes, maps are thresholded at *P*<0.001 uncorrected and a cluster extent of *k*=20. (**b**) Scatterplot for illustration. FWE, family-wise error.

First, no differences were observed between low- and high-impulsive groups at a whole-brain level nor when looking at anatomical or fMRI-derived regions of interest. Second, a significant positive correlation between dorsolateral prefrontal gray matter density and parameter *ω* (a higher *ω* indicates more model-based choices) was observed (Montreal Neurological Institute *x*=−42, *y*=22, *z*=50, *t*=5.04, p-FWE=0.05 for bilateral medial and lateral PFC, *r*=0.59, *R*^2^=0.35, 95% confidence interval (0.37, 0.75, Figure 4).

## Discussion

The present study shows high trait impulsivity in healthy individuals to be accompanied by behavioral and neural signatures in favor of a model-free system of behavioral control. Although we did not observe a shift in the balance of behavioral control toward model-free control in high-impulsive individuals, two main findings support this notion: first, in line with behavioral raw data analysis, computational modeling revealed a subtle but significant accentuation of model-free control in high-impulsive individuals; second, lateral prefrontal model-based signals were reduced in high-impulsive individuals.

### Trait impulsivity, behavioral control and addiction

High-impulsive individuals showed an accentuation of a model-free control system, namely the impact of reward prediction errors on first-stage decision values was elevated. In contrast to addicted and other psychiatric patient samples,^7, 20^ we did not find evidence for an impairment of model-based behavioral control in our sample of high-impulsive individuals. Utilizing the same sequential decision task, it was recently demonstrated that patients with addictive disorders and other conditions from the impulsivity–compulsivity spectrum show a shift of behavioral control from model-based toward model-free control.^7, 20^ In both patient studies,^7, 20^ model-based control was reduced (reward × state × group interaction or lower parameter *ω*) but patients did not differ from controls regarding measures of the model-free system alone (reward × group interaction or higher parameter *λ*). So far, the origin of behavioral findings in patients remains unclear: they could result from an antecedent accentuation in a model-free system ultimately reducing model-based control, although this is not supported by hitherto existing studies; they could be linked to an arbitration or integration problem between two systems or they could be tied to impairments of a model-based system alone. Studies suggest that interindividual variability in cognitive capacities relate to a model-based system.^33, 40, 41, 42^ Interestingly, Sebold *et al.*^20^ showed in alcohol-dependent patients that reduced model-based control was at least abolished when correcting for cognitive capacities and similar control analyses were not reported in Voon *et al.*^7^ Here, we show that the risk factor impulsivity results in an accentuation within a model-free control system alone, although—unlike in addiction and other patients groups—an overall balance of control was not altered. Importantly, general cognition is very unlikely to account for the findings in the present study. Nonetheless, it remains an intriguing question why some healthy individuals perform this task in a model-free way. One line of reasoning includes that ongoing model-based evaluation during the main experiment challenges limited computational resources.^14^ Studies in healthy individuals support this view by showing that interindividual differences in cognitive capacities, in particular working memory,^40, 41, 42^ relate to the balance of model-free and model-based controls in this task. Further associations were shown for acute stress reactivity and chronic stress levels^41, 43^ as well as striatal presynaptic dopamine levels.^19^ Another idea involves that such individuals could have a ‘false' model or a ‘false belief' about the state transition, for example, a subjective illusion of control. Interestingly, a recent study reported that healthy adults with a subjective belief of control over reward delivery, which was objectively not given, showed increased ventral striatal and lateral prefrontal activation during reward anticipation.^44^ However, this idea cannot be adequately tested with the task applied in the present study and instead requires experimental designs specifically tailored to address this. Together, these factors most likely also have an important role in explaining the emergence of a dominance of model-free control in psychopathological groups performing this task.^7, 20^ With respect to addictive behaviors, it is conceivable that longitudinal interactions with acute drug abuse,^45^ chronic drug consumption^46^ or acute and chronic stress^41, 43^ may finally prompt a pattern of reduced model-based control and leave model-free control as the only available mode of control in patients.

### Reduced lateral prefrontal model-based signatures in high-impulsive individuals

High-impulsive individuals exhibited reduced model-based signatures in a sector of the lateral PFC. In previous research, measures of impulsivity were linked to inferior parts of lateral PFC,^47, 48^ which is an important region exhibiting top–down control^49^ also during sequential decision-making.^38^ Indeed, it was proposed that altered behavioral control in addiction and impulsivity is associated or even results from reduced prefrontal top–down control exerted over striatal regions.^1^ In the present study, reduced model-based signatures in the lateral PFC of high-impulsive individuals could indicate such deficient top–down control. In line, Daw *et al.*^18^ suggested that a covariation of ventral striatal activation with model-free but most strikingly also model-based signatures could result from a top–down, prefrontal to striatal, information flow between the two control systems. However, it remains an important question what precisely determines the degree of control exerted over striatal regions, given that ventral striatal model-based signatures remained unaffected in high-impulsive individuals. One potential explanation for unaffected ventral striatal signals, and also intact model-based behavioral control, is that medial PFC model-based signatures did not differ between high- and low-impulsive groups. Given a likely role of medial PFC in integrating decision values from both systems,^38, 50^ intact model-based coding in medial PFC may preserve neural top–down control and thus behavioral model-based control. A failure of this medial PFC function may ultimately result in an overall shift of behavioral control as observed behaviorally in patients.^7, 20^

Dopamine was also suggested to have an important role in modulating top–down control in frontostriatal circuits.^51, 52, 53^ Whereas blunted (ventral) striatal dopamine function was reported in addicted patients both pre- and postsynaptically,^54, 55, 56, 57, 58^ animal research has shown that Positron emission tomography (PET) measures of ventral striatal dopamine D2 receptor availability are lower in high-impulsive, stimulant-naive rats and predict escalated levels of stimulant self-administration.^11^ Interestingly, in human PET studies, higher levels of impulsivity were shown to be mediated by lower levels of presynaptic dopamine function.^59, 60^ Using the same task and analytic strategy as in the present study, pharmacological elevation of presynaptic dopamine induced a bias toward model-based choices.^31^ This positive association between model-based control and dopamine was confirmed in a human PET-fMRI study with respect to ventral striatal presynaptic dopamine levels.^19^ Interestingly, in the latter study ventral striatal presynaptic dopamine levels were also shown to be positively correlated with model-based signatures in lateral PFC^19^ at nearby coordinates where model-based signatures were found to be reduced in high-impulsive individuals in the present study. Although low dopamine levels appear to be associated with reduced model-based control, impulsivity and vulnerability to addiction, the exact interplay of these variables still remains to be elucidated in future translational and longitudinal studies.

One may further speculate that a lateral PFC dysfunction characterizes the impulsive spectrum. Indeed, the observed reduction of model-based signals in the lateral PFC nicely matches endophenotype studies that revealed lateral PFC (in particular inferior frontal gyrus) as a vulnerability nexus in siblings of stimulant-dependent patients with regard to white matter integrity and gray matter density.^6^ Reduced structural PFC integrity was not observed in our sample of high-impulsive individuals, which may be due to differences in sample characteristics. In particular, high-impulsive siblings of addicted patients also show cognitive impairments.^6^ To isolate effects of impulsivity, we explicitly choose to study high-impulsive healthy individuals who did not show differences in cognitive measures when compared with low-impulsive individuals. Notably, all behavioral and fMRI results associated with high impulsivity were independent of individual variability in these cognitive measures or gray matter density. Irrespectively of impulsivity, dorsolateral prefrontal gray matter density was positively related to a balance of model-free and model-based control. This confirms previous findings linking prefrontal gray matter density to a balance of control, albeit in a different prefrontal area.^7^

Thus and taken together so far, it is likely that multiple ‘hits' on the functional and structural level multiplex to a vulnerability pattern for addiction.^61^

### Limitations

The presented behavioral and neural results warrant replication and their predictive relevance remains an important target for future longitudinal studies. Thus, future studies should follow up healthy participants from extreme ends of personality traits, at-risk samples and patients to examine whether alterations in behavioral control predict future development of drug intake as suggested by animal models.^11^ Regarding decision-making tasks that aim to assess model-free habitual and model-based goal-directed behavior, construct validity remains an important issue. Work from our group has studied construct validity by testing the applied version of sequential decision-making and a selective devaluation task in the same participants.^62^ Indeed, although in a limited sample size, a positive correlation between the main outcome measures of both tasks was found.^62^ A similar observation has recently been confirmed in a larger sample and a different design incorporating devaluation into the sequential decision task.^63^ Although more indirectly, the by now repeatedly reported association between model-based control and general cognitive capacities^40, 41, 42, 64^ also supports construct validity of the applied task in terms of the computational costs, and thus higher cognitive demands, of model-based control.

## Conclusion

We believe we present first evidence for the idea that high impulsivity in healthy individuals is accompanied by behavioral and neural signatures in favor of model-free behavioral control. The behavioral results in healthy high-impulsive individuals were qualitatively different to findings in patients with the same task. Effects of smoking, alcohol intake, general cognition or structural brain measures did not account for the findings. Adopting a *Computational Psychiatry* approach, we show that these techniques represent feasible and mechanistically informative tools that may enrich future longitudinal studies.^21, 65^

## Figures and Tables

**Table 1:** Sample characteristics

*Healthy participants*	*With high trait impulsivity (*N=*24)*	*With low trait impulsivity (*N=*26)*	*P-value*
Age (years; 24/26)	27.29±3.67 (22–33)	27.58±3.74 (20–33)	0.78
Gender (24/26)	12 Female/12 male	13 Female/13 male	
BIS total (24/26)	74.76±5.07 (68–90)	50.31±3.78 (41–58)	<0.001
Smoking (24/26)	1 Smoker	1 Smoker	
Drinking (g; 24/25)	23.48±21.28 (0–74)	17.48±14.84 (0–63)	0.26
Verbal intelligence (24/25)	109.88±7.60 (97–129)	113.08±5.93 (104–122)	0.11
Working memory (24/25)	8.08±2.10 (4–12)	8.12±1.97 (5–12)	0.82
Processing speed (24/25)	88.26±11.29 (4–12)	82.71±11.45 (4–12)	0.09

Abbreviation: BIS, Barratt Impulsiveness Scale.

Group means with s.d.'s and range in brackets are reported; for group comparisons two-sample *t*-tests were used.

## References

[bib1] Dalley JW, Everitt BJ, Robbins TW. Impulsivity, compulsivity, and top-down cognitive control. Neuron 2011; 69: 680–694. 21338879 10.1016/j.neuron.2011.01.020

[bib2] Robbins TW, Gillan CM, Smith DG, de Wit S, Ersche KD. Neurocognitive endophenotypes of impulsivity and compulsivity: towards dimensional psychiatry. Trends Cogn Sci 2012; 16: 81–91. 22155014 10.1016/j.tics.2011.11.009

[bib3] Verdejo-Garcia A, Lawrence AJ, Clark L. Impulsivity as a vulnerability marker for substance-use disorders: review of findings from high-risk research, problem gamblers and genetic association studies. Neurosci Biobehav Rev 2008; 32: 777–810. 18295884 10.1016/j.neubiorev.2007.11.003

[bib4] Ersche KD, Turton AJ, Pradhan S, Bullmore ET, Robbins TW. Drug addiction endophenotypes: impulsive versus sensation-seeking personality traits. Biol Psychiatry 2010; 68: 770–773. 20678754 10.1016/j.biopsych.2010.06.015 PMC3485555

[bib5] Ersche KD, Jones PS, Williams GB, Smith DG, Bullmore ET, Robbins TW. Distinctive personality traits and neural correlates associated with stimulant drug use versus familial risk of stimulant dependence. Biol Psychiatry 2013; 74: 137–144. 23273722 10.1016/j.biopsych.2012.11.016 PMC3705207

[bib6] Ersche KD, Jones PS, Williams GB, Turton AJ, Robbins TW, Bullmore ET. Abnormal brain structure implicated in stimulant drug addiction. Science 2012; 335: 601–604. 22301321 10.1126/science.1214463

[bib7] Voon V, Derbyshire K, Ruck C, Irvine MA, Worbe Y, Enander J et al. Disorders of compulsivity: a common bias towards learning habits. Mol Psychiatry 2014; 20: 345–352. 24840709 10.1038/mp.2014.44 PMC4351889

[bib8] de Wit S, Watson P, Harsay HA, Cohen MX, van de Vijver I, Ridderinkhof KR. Corticostriatal connectivity underlies individual differences in the balance between habitual and goal-directed action control. J Neurosci 2012; 32: 12066–12075. 22933790 10.1523/JNEUROSCI.1088-12.2012 PMC6621537

[bib9] Everitt BJ, Belin D, Economidou D, Pelloux Y, Dalley JW, Robbins TW. Review. Neural mechanisms underlying the vulnerability to develop compulsive drug-seeking habits and addiction. Philos Trans R Soc Lond B Biol Sci 2008; 363: 3125–3135. 18640910 10.1098/rstb.2008.0089 PMC2607322

[bib10] Hogarth L, Balleine BW, Corbit LH, Killcross S. Associative learning mechanisms underpinning the transition from recreational drug use to addiction. Ann N Y Acad Sci 2013; 1282: 12–24. 23126270 10.1111/j.1749-6632.2012.06768.x

[bib11] Dalley JW, Fryer TD, Brichard L, Robinson ES, Theobald DE, Laane K et al. Nucleus accumbens D2/3 receptors predict trait impulsivity and cocaine reinforcement. Science 2007; 315: 1267–1270. 17332411 10.1126/science.1137073 PMC1892797

[bib12] Dickinson AD. Action and habits: the development of behavioural autonomy. Philos Trans R Soc Lond B Biol Sci 1985; 308: 67–78.

[bib13] Dolan RJ, Dayan P. Goals and habits in the brain. Neuron 2013; 80: 312–325. 24139036 10.1016/j.neuron.2013.09.007 PMC3807793

[bib14] Daw ND, Niv Y, Dayan P. Uncertainty-based competition between prefrontal and dorsolateral striatal systems for behavioral control. Nat Neurosci 2005; 8: 1704–1711. 16286932 10.1038/nn1560

[bib15] Dayan P. Dopamine, reinforcement learning, and addiction. Pharmacopsychiatry 2009; 42: S56–S65. 19434556 10.1055/s-0028-1124107

[bib16] Balleine BW, Dickinson A. Goal-directed instrumental action: contingency and incentive learning and their cortical substrates. Neuropharmacology 1998; 37: 407–419. 9704982 10.1016/s0028-3908(98)00033-1

[bib17] Doll BB, Simon DA, Daw ND. The ubiquity of model-based reinforcement learning. Curr Opin Neurobiol 2012; 22: 1075–1081. 22959354 10.1016/j.conb.2012.08.003 PMC3513648

[bib18] Daw ND, Gershman SJ, Seymour B, Dayan P, Dolan RJ. Model-based influences on humans' choices and striatal prediction errors. Neuron 2011; 69: 1204–1215. 21435563 10.1016/j.neuron.2011.02.027 PMC3077926

[bib19] Deserno L, Huys Q, Boehme R, Buchert R, Heinze HJ, Grace AA et al. Ventral striatal presynaptic dopamine reflects behavioral and neural signatures of model-based control during sequential decision-making. Proc Natl Acad Sci USA 2015; 112: 1595–1600. 25605941 10.1073/pnas.1417219112 PMC4321318

[bib20] Sebold M, Deserno L, Nebe S, Schad DJ, Garbusow M, Hagele C et al. Model-based and model-free decisions in alcohol dependence. Neuropsychobiology 2014; 70: 122–131. 25359492 10.1159/000362840

[bib21] Montague PR, Dolan RJ, Friston KJ, Dayan P. Computational psychiatry. Trends Cogn Sci 2012; 16: 72–80. 22177032 10.1016/j.tics.2011.11.018 PMC3556822

[bib22] Stephan KE, Mathys C. Computational approaches to psychiatry. Curr Opin Neurobiol 2014; 25: 85–92. 24709605 10.1016/j.conb.2013.12.007

[bib23] Wang XJ, Krystal JH. Computational psychiatry. Neuron 2014; 84: 638–654. 25442941 10.1016/j.neuron.2014.10.018 PMC4255477

[bib24] Hogarth L, Chase HW, Baess K. Impaired goal-directed behavioural control in human impulsivity. Quart J Exp Psychol 2012; 65: 305–316. 10.1080/17470218.2010.518242 PMC3471322 21077008

[bib25] Patton JH, Stanford MS, Barratt ES. Factor structure of the Barratt impulsiveness scale. J Clin Psychol 1995; 51: 768–774. 8778124 10.1002/1097-4679(199511)51:6<768::aid-jclp2270510607>3.0.co;2-1

[bib26] Stanford MS, Mathias CW, Dougherty DM, Lake SL, Anderson NE, Patton JH. Fifty years of the Barratt Impulsiveness Scale: an update and review. Pers Individ Dif 2009; 47: 385–395.

[bib27] First MB, Spitzer RL, Gibbon M, Williams J. Structured Clinical interview for DSM-IV-TR Axis I Disorders, Research Version, Patient Edition With Psychotic Screen (SCID-I/P W/ PSY SCREEN). New York State Psychiatric Institute: New York, NY, USA, 2001.

[bib28] Schmidt K-H, Metzler P. Wortschatztest (WST). Beltz Test GmbH: Weinheim, 1992.

[bib29] Wechsler D. Wechsler Adult Intelligence Scale Manual. Psychological Corporation: New York, NY, USA, 1955.

[bib30] Linda CS, Mark BS. Timeline follow-back: a technique for assessing self-reported alcohol consumption In: Litten RZ et al Measuring Alcohol Consumption: Psychosocial and Biological Methods. Humana: New Jersey, NY, USA, 1992.

[bib31] Wunderlich K, Smittenaar P, Dolan RJ. Dopamine enhances model-based over model-free choice behavior. Neuron 2012; 75: 418–424. 22884326 10.1016/j.neuron.2012.03.042 PMC3417237

[bib32] Eppinger B, Walter M, Heekeren HR, Li SC. Of goals and habits: age-related and individual differences in goal-directed decision-making. Front Neurosci 2013; 7: 253. 24399925 10.3389/fnins.2013.00253 PMC3871973

[bib33] Smittenaar P, FitzGerald TH, Romei V, Wright ND, Dolan RJ. Disruption of dorsolateral prefrontal cortex decreases model-based in favor of model-free control in humans. Neuron 2013; 80: 914–919. 24206669 10.1016/j.neuron.2013.08.009 PMC3893454

[bib34] Sutton RS, Barto AG. Reinforcement Learning: An Introduction. MIT Press: : Cambridge, MA, USA, 1998.

[bib35] Stephan KE, Penny WD, Daunizeau J, Moran RJ, Friston KJ. Bayesian model selection for group studies. Neuroimage 2009; 46: 1004–1017. 19306932 10.1016/j.neuroimage.2009.03.025 PMC2703732

[bib36] Ashburner J, Friston KJ. Unified segmentation. Neuroimage 2005; 26: 839–851. 15955494 10.1016/j.neuroimage.2005.02.018

[bib37] Glascher J, Daw N, Dayan P, O'Doherty JP. States versus rewards: dissociable neural prediction error signals underlying model-based and model-free reinforcement learning. Neuron 2010; 66: 585–595. 20510862 10.1016/j.neuron.2010.04.016 PMC2895323

[bib38] Lee SW, Shimojo S, O'Doherty JP. Neural computations underlying arbitration between model-based and model-free learning. Neuron 2014; 81: 687–699. 24507199 10.1016/j.neuron.2013.11.028 PMC3968946

[bib39] Tzourio-Mazoyer N, Landeau B, Papathanassiou D, Crivello F, Etard O, Delcroix N et al. Automated anatomical labeling of activations in SPM using a macroscopic anatomical parcellation of the MNI MRI single-subject brain. Neuroimage 2002; 15: 273–289. 11771995 10.1006/nimg.2001.0978

[bib40] Otto AR, Gershman SJ, Markman AB, Daw ND. The curse of planning: dissecting multiple reinforcement-learning systems by taxing the central executive. Psychol Sci 2013; 24: 751–761. 23558545 10.1177/0956797612463080 PMC3843765

[bib41] Otto AR, Raio CM, Chiang A, Phelps EA, Daw ND. Working-memory capacity protects model-based learning from stress. Proc Natl Acad Sci USA 2013; 110: 20941–20946. 24324166 10.1073/pnas.1312011110 PMC3876216

[bib42] Schad DJ, Junger E, Sebold M, Garbusow M, Bernhardt N, Javadi AH et al. Processing speed enhances model-based over model-free reinforcement learning in the presence of high working memory functioning. Front Psychol 2014; 5: 1450. 25566131 10.3389/fpsyg.2014.01450 PMC4269125

[bib43] Radenbach C, Reiter AMF, Engert V, Sjoerds Z, Villringer A, Heinze HJ et al. The interaction of acute and chronic stress impairs model-based behavioral control. Psychoneuroendocrinology 2015; 53: 268–280. 25662093 10.1016/j.psyneuen.2014.12.017

[bib44] Lorenz RC, Gleich T, Kuhn S, Pohland L, Pelz P, Wustenberg T et al. Subjective illusion of control modulates striatal reward anticipation in adolescence. Neuroimage 2015; 117: 250–257. 25988224 10.1016/j.neuroimage.2015.05.024

[bib45] Hogarth L, Attwood AS, Bate HA, Munafo MR. Acute alcohol impairs human goal-directed action. Biol Psychol 2012; 90: 154–160. 22406757 10.1016/j.biopsycho.2012.02.016

[bib46] Deserno L, Beck A, Huys Q, Lorenz R, Buchert R, Buchholz HG et al. Chronic alcohol intake abolishes the relationship between dopamine synthesis capacity and learning signals in ventral striatum. Eur J Neurosci 2015; 41: 477–486. 25546072 10.1111/ejn.12802 PMC4455879

[bib47] Wilbertz T, Deserno L, Horstmann A, Neumann J, Villringer A, Heinze HJ et al. Response inhibition and its relation to multidimensional impulsivity. Neuroimage 2014; 103C: 241–248. 10.1016/j.neuroimage.2014.09.021 25241087

[bib48] Farr OM, Hu S, Zhang S, Li CS. Decreased saliency processing as a neural measure of Barratt impulsivity in healthy adults. Neuroimage 2012; 63: 1070–1077. 22885245 10.1016/j.neuroimage.2012.07.049 PMC3472158

[bib49] Koechlin E, Ody C, Kouneiher F. The architecture of cognitive control in the human prefrontal cortex. Science 2003; 302: 1181–1185. 14615530 10.1126/science.1088545

[bib50] Wunderlich K, Dayan P, Dolan RJ. Mapping value based planning and extensively trained choice in the human brain. Nat Neurosci 2012; 15: 786–791. 22406551 10.1038/nn.3068 PMC3378641

[bib51] Cools R. Dopaminergic control of the striatum for high-level cognition. Curr Opin Neurobiol 2011; 21: 402–407. 21531543 10.1016/j.conb.2011.04.002

[bib52] Seamans JK, Yang CR. The principal features and mechanisms of dopamine modulation in the prefrontal cortex. Prog Neurobiol 2004; 74: 1–58. 15381316 10.1016/j.pneurobio.2004.05.006

[bib53] Braver TS, Cohen JD. Dopamine, cognitive control, and schizophrenia: the gating model. Prog Brain Res 1999; 121: 327–349. 10551035 10.1016/s0079-6123(08)63082-4

[bib54] Martinez D, Gil R, Slifstein M, Hwang DR, Huang Y, Perez A et al. Alcohol dependence is associated with blunted dopamine transmission in the ventral striatum. Biol Psychiatry 2005; 58: 779–786. 16018986 10.1016/j.biopsych.2005.04.044

[bib55] Martinez D, Narendran R, Foltin RW, Slifstein M, Hwang DR, Broft A et al. Amphetamine-induced dopamine release: markedly blunted in cocaine dependence and predictive of the choice to self-administer cocaine. Am J Psychiatry 2007; 164: 622–629. 17403976 10.1176/ajp.2007.164.4.622

[bib56] Martinez D, Saccone PA, Liu F, Slifstein M, Orlowska D, Grassetti A et al. Deficits in dopamine D(2) receptors and presynaptic dopamine in heroin dependence: commonalities and differences with other types of addiction. Biol Psychiatry 2012; 71: 192–198. 22015315 10.1016/j.biopsych.2011.08.024 PMC3253988

[bib57] Heinz A, Siessmeier T, Wrase J, Hermann D, Klein S, Grusser SM et al. Correlation between dopamine D(2) receptors in the ventral striatum and central processing of alcohol cues and craving. Am J Psychiatry 2004; 161: 1783–1789. 15465974 10.1176/appi.ajp.161.10.1783

[bib58] Volkow ND, Wang GJ, Fowler JS, Logan J, Hitzemann R, Ding YS et al. Decreases in dopamine receptors but not in dopamine transporters in alcoholics. Alcohol Clin Exp Res 1996; 20: 1594–1598. 8986209 10.1111/j.1530-0277.1996.tb05936.x

[bib59] Buckholtz JW, Treadway MT, Cowan RL, Woodward ND, Li R, Ansari MS et al. Dopaminergic network differences in human impulsivity. Science 2010; 329: 532. 20671181 10.1126/science.1185778 PMC3161413

[bib60] Schluter T, Winz O, Henkel K, Prinz S, Rademacher L, Schmaljohann J et al. The impact of dopamine on aggression: an [18 F]-FDOPA PET Study in healthy males. J Neurosci 2013; 33: 16889–16896. 24155295 10.1523/JNEUROSCI.1398-13.2013 PMC6618436

[bib61] Heinz AJ, Beck A, Meyer-Lindenberg A, Sterzer P, Heinz A. Cognitive and neurobiological mechanisms of alcohol-related aggression. Nat Rev Neurosci 2011; 12: 400–413. 21633380 10.1038/nrn3042

[bib62] Friedel E, Koch SP, Wendt J, Heinz A, Deserno L, Schlagenhauf F. Devaluation and sequential decisions: linking goal-directed and model-based behavior. Front Hum Neurosci 2014; 8: 587. 25136310 10.3389/fnhum.2014.00587 PMC4120761

[bib63] Gillan CM, Otto AR, Phelps EA, Daw ND. Model-based learning protects against forming habits. Cogn Affect Behav Neurosci 2015; 15: 523–536. 25801925 10.3758/s13415-015-0347-6 PMC4526597

[bib64] Otto AR, Skatova A, Madlon-Kay S, Daw ND. Cognitive control predicts use of model-based reinforcement learning. J Cogn Neurosci 2015; 27: 319–333. 25170791 10.1162/jocn_a_00709 PMC4387848

[bib65] Brodersen KH, Deserno L, Schlagenhauf F, Lin Z, Penny WD, Buhmann JM et al. Dissecting psychiatric spectrum disorders by generative embedding. NeuroImage 2014; 4: 98–111. 24363992 10.1016/j.nicl.2013.11.002 PMC3863808

