# Supplementary material for: Lateral prefrontal model-based signatures are reduced in healthy individuals with high trait impulsivity
Source: Transl Psychiatry. 2015 Oct 13;5(10):e659–. doi: 10.1038/tp.2015.139 (PMC4930122; doi:10.1038/tp.2015.139)
Supplement: Supplementary Information [file tp2015139x1.doc]

Supplementary Information for:

**Lateral prefrontal model-based signals are reduced in healthy individuals with high trait impulsivity**

*Running Title: Trait impulsivity and model-free versus model-based control*

Dr. Lorenz Deserno^1,2,3^, cand-med. Tilmann Wilbertz^1^, Dipl-Psych. Andrea Reiter^1,4^, Dr. Annette Horstmann^5,6^, Dr. Jane Neumann^5,6^, Prof. Dr. Arno Villringer^5,6,7,8^, Prof. Dr. Hans-Jochen Heinze^1,3,9^, Dr. Florian Schlagenhauf^1,2^

*^1^ Max Planck Fellow Group ‘Cognitive and Affective Control of Behavioral Adaptation’, Max Planck Institute for Human Cognitive and Brain Sciences, Leipzig, Germany*

*^2^ Department of Psychiatry and Psychotherapy, Campus Charité Mitte, Charité - Universitätsmedizin Berlin, Germany*

*^3^ Department of Neurology, Otto-von-Guericke University, Magdeburg, Germany*

*^4^ International Max Planck Research School on the Neuroscience of Communication (IMPRS NeuroCom), Leipzig, Germany*

*^5^ Department of Cognitive Neurology, Max Planck Institute for Human Cognitive and Brain Sciences, Leipzig, Germany*

*^6^ IFB AdiposityDiseases, University of Leipzig, Germany*

*^7^ Clinic for Cognitive Neurology, University Hospital Leipzig, Leipzig, Germany*

*^8^ Berlin School of Mind & Brain and Mind and Brain Institute, Humboldt University, Berlin, Germany*

*^9^ Department of Behavioral Neurology, Leibniz Institute for Neurobiology, Magdeburg, Germany*

Contact Information:

Dr. Lorenz Deserno

Max Planck Institute for Human Cognitive and Brain Sciences

Tel. +49-341-9940 109

Fax. +49-341-9940 2221

E-mail: [deserno@cbs.mpg.de](mailto:deserno@cbs.mpg.de)

**Supplementary Methods**

Computational model. As in previous studies^1-4^, we adopted a modeling approach to disentangle influences of model-free and model-based control on participant’s choice behavior. As described in the main manuscript, three types of algorithms were applied. All three algorithms learn values (Q) for each of the stimuli, which appear in the task as three pairs (s_A_, s_B_, s_C_). s_A_ refers to the first-stage stimuli where values of model-free and model-based algorithms differ. s_B_ and s_C_ refer to the two pairs of second-stage stimuli. In the following equations, a indexes the chosen stimulus and index i denotes the stage (i=1 for S_A_ at the first stage and i=2 for S_B_ or S_C_ at the second stage) and t signifies the trial.

First, the model-free algorithm was SARSA(λ) which learns values *retrospectively* after prediction errors occurred^5^:

[1]
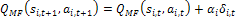


[2]
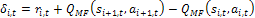


Here, α denotes learning rates for the first and second stage. Notably,
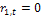
 because no reward is delivered after a first-stage choice and
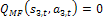
 because the task has only two states. Further, we allow for an additional stage-skipping update of first-stage values by introducing the parameter λ. As part of the model-free algorithm, this parameter connects the two stages in way that reward prediction errors at the second stage can influence first-stage values:

[3]
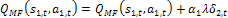


Importantly, λ accounts for the main effect of reward as observed in the analysis of first-stage stay-switch behavior but not for an interaction of reward and state. Notably, a model-free temporal-difference algorithm, here SARSA(λ), could acquire the state transition given enough time and stationary reward probabilities at the second stage. In the applied task, second-stage rewards probabilities changed slowly and independently according Gaussian random walks, which were identical to Daw et al. (2011)^1^.

Second, the model-based algorithm learns values *prospectively* and computes first-stage values by multiplying the maximum values at the second stage with the explicitly instructed transition probabilities:

[4]
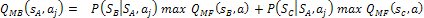


In equation 4, second-stage values come from the model-free algorithm because the model-based algorithm converges with the model-free algorithm at the second stage. Note that this approach simplifies transition learning as this algorithm does not learn transition probabilities incrementally but this is in line with the task instructions and the training. In a simulation by Daw et al. (2011) it could be shown that this approach outperforms incremental learning of the transition probabilities; moreover, this was identically applied in other non-clinical^3, 4^ and clinical studies^2^.

Third, the hybrid algorithm connects model-free and model-based values via the weighting parameter ω:

[5]
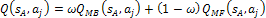


Importantly, ω gives a weighing of the relative influence of model-free and model-based values. It represents the balance of the two control strategies.

Finally, we transform values into action probabilities using a softmax for values Q:

[6]
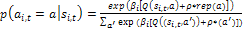


Here, β controls the stochasticity of the choices at the first and second stage separately. The additional parameter ρ captures first-stage choice perseveration and rep is an indicator function that equals 1 if the previous first-stage choice was the same^1^.

Model fitting. Constrained parameters were transformed to a logistic (α, λ, ω) or exponential (β) distribution to enforce constraints and to render normally distributed parameter estimates. To infer the maximum-a-posteriori estimate *MAP* of parameters θ, we use a Gaussian prior with mean and variance μ and σ:

[7]
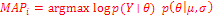


where Y represents the data in terms of actions A_i_ per subject *i*. We set priors empirically to the maximum likelihood estimates *ML* of μ and σ given the data by all subjects:

[8]
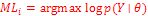


and achieve this by using Expectation-Maximisation. For an in-depth description please compare^6, 7^. Inferred parameters were distributed similarly as observed in previous studies with the same task e.g^. 1, 2-4, 8^ (Table S-2). All modeling analyses were performed using Matlab 2010b. Code of the analyses is available from the authors upon request.

Model comparison. For all three models, we first report the negative log-likelihood and the Bayesian Information Criterion (BIC) based on the negative log-likelihood (Table S1). Second, we approximate the model evidence by integrating out the free parameters. The integral was approximated by sampling from the prior distribution and we therefore add the subscript ‘int’ to the BIC (Table S1; compare ^6, 7^). Third and reported in the main manuscript, we submit this integrated likelihood to a random-effects Bayesian model selection procedure^9^ (spm_BMS contained in SPM8). We also show that best-fitting parameters nicely reproduce the observed behavior.

Relationship of the parameters ω and λ. In parallel to the analysis of first-state choice data, the parameter λ resembles the main effect of reward on first-stage behavior while ω relates to the interaction of reward and state. However, relatively low levels of ω could either result from a reduced influence of first-stage model-based values or from a stronger weighting of first-stage model-free values. Thus at certain levels of λ, ω will decrease and vice versa. Given relatively midrange levels for both parameters in a sample, no correlation would be expected.

First, as we have previously published independent samples with this task including the identical modeling analysis, we did check the correlation of both parameters in all three samples^4, 8^ (for the former from the control condition only): Deserno et al. (2015)^4^: r=-.09, p=.65, mean ω=.53±.18 (SD), λ.71±.09; Radenbach et al. (2015)^8^: r=-.41, p=.01, mean ω=.68±.08, λ**=**.57±.11; presented study: r=-.34, p=.02, ω=.65±.09, λ**=**.60±.11; These correlations obtained across three independent samples demonstrate that a consistent correlation between the two parameters cannot be assumed. Importantly, the correlation in the present sample was not driven by one of the two groups (low-impulsivity r=-.40, p=.05, mean ω=.59±.11, λ**=**.62±.10, high-impulsivity r=-.39, p=.06, mean ω=.61±.11, λ**=**.69±.08). Given this relatively low to moderate correlations, we conclude that a change in one of the two does not simply imply a change in the other, which would in fact render an additional parameter redundant. This is also supported by the observation that the parameter ω can be well re-fitted from generated data based on the inferred parameters (please compare^4^).

In the same vein, we were asked whether fitting λ as a free parameter could have concealed a group difference on ω. In fact, finding a difference on ω when fixing λ would be surprising because the raw data does not support a change in the overall balance of model-free and model-based control (while the reward x impulsivity in raw data supports an effect of impulsivity on λ). Thus, such an effect due to fixing λ would speak for redundancy of the two parameters, which is, as pointed out, not the case. In line with this reasoning, the suggested analysis did not reveal any difference in ω when keeping λ fixed to the sample mean of .65 and fitting the model (ω high-impulsive .61±.11, low-impulsive .60±.11, T(1,48)=.38, p=.70).

**Supplementary Results**

***Table S1 Model Selection.*** *-LL: negative log-likelihood; BIC: Bayesian Information Criterion, the subscript int refers to integrating out the free parameters; XP: Exceedance Probability; all=all participants n=50, high=high-impulsive n=24, low=low-impulsive n=26.*

|  |  | -LL | BIC | BIC_int_ | XP |
| --- | --- | --- | --- | --- | --- |
| hybrid with λ | all | 9261.94 | 18593.14 | 19326.75 | .9999 |
|  | high | 4578.76 | 9221.65 | 9560.81 | .9950 |
|  | low | 4683.18 | 9431.04 | 9825.49 | .9997 |
|  |  | Δ -LL hybrid | Δ BIC hybrid | Δ BIC_int_ hybrid |  |
| hybrid without λ | all | -151.14 | 292.39 | 265.54 | 0 |
|  | high | -83.09 | 157.01 | 147.86 | 3e-04 |
|  | low | -68.05 | 126.87 | 109.17 | 1e-05 |
| model-based | all | -260.38 | 491.07 | 325.59 | 0 |
|  | high | -114.20 | 200.92 | 128.38 | 9e-05 |
|  | low | -146.18 | 264.63 | 171.68 | 3e-05 |
| model-free with λ | all | -304.58 | 599.27 | 468.23 | 1e-05 |
|  | high | -120.77 | 232.37 | 183.13 | .0050 |
|  | low | -183.81 | 358.38 | 276.60 | .0003 |
| model-free without λ | all | -549.17 | 1078.54 | 905.82 | 0 |
|  | high | -247.29 | 476.26 | 403.75 | 0 |
|  | low | -301.88 | 585.27 | 485.05 | 1e-05 |

**Table S2 Distribution of best-fitting parameters.** Hybrid Model. : weighting of model-free and model-based values; α1,α1: learning rates at the first and second stage; : stage-skipping update; Softmax Observation Model. β1,β2: stochasticity of first- and second-stage choices; -LL: negative log-likelihood; all=all participants n50, high=high-impulsive n=24, low=low-impulsive n=26.

|  |  |  | α_1_ | α_2_ |  | β_1_ | β_2_ |  | -LL |
| --- | --- | --- | --- | --- | --- | --- | --- | --- | --- |
| 25^th^ percentile | all | .55 | .36 | .49 | .60 | 4.28 | 2.21 | .10 | 222.24 |
|  | high | .57 | .41 | .43 | .63 | 3.76 | 2.15 | .13 | 227.61 |
|  | low | .53 | .30 | .55 | .55 | 5.01 | 2.34 | .08 | 206.64 |
| Median | all | .60 | .49 | .62 | .65 | 7.20 | 3.08 | .14 | 179.47 |
|  | high | .62 | .52 | .63 | .69 | 6.13 | 3.04 | .15 | 191.16 |
|  | low | .59 | .40 | .62 | .64 | 7.62 | 3.21 | .12 | 176.77 |
| 75^th^ percentile | all | .68 | .65 | .71 | .71 | 8.46 | 3.89 | .18 | 141.66 |
|  | high | .70 | .66 | .74 | .73 | 7.99 | 3.85 | .18 | 149.60 |
|  | low | .68 | .60 | .68 | .68 | 9.28 | 3.89 | .18 | 141.62 |

**Table S3 fMRI results.** Whole-brain results for the conjunction of model-free and model-based learning signals across both groups (n=48, 23 high-impulsive and 25 low-impulsive participants).

| *region* | | | *coordinates* | *t-value* | *p-FWE*  *peak-level* | *k* | *p-FWE*  *cluster-level* |
| --- | --- | --- | --- | --- | --- | --- | --- |
|  | **Conjunction of model-free and model-based** | | | | | | |
| ***medial PFC*** | | 0, 50, 0 | | 4.83 | .059 | 919 | 2.55e-05 |
|  |  | 0, 44, 8 | | 4.59 | .125 |  |  |
|  |  | -4, 30, 8 | | 4.02 | .545 |  |  |
| ***ventral striatum*** | | -12, 12, -8 | | 5.51 | .005 | 1451 | 2.49e-07 |
|  | | 12, 8, -8 | | 5.42 | .007 |  |  |
|  |  | -8, 12, 2 | | 5.23 | .014 |  |  |
| ***lateral PFC/OFC*** | | 20, 28, -16 | | 4.43 | .200 | 250 | 0.036 |
|  |  | 38, 24, -16 | | 3.98 | .587 |  |  |
|  |  | 32,42, -14 | | 3.77 | .796 |  |  |
| ***lateral parietal*** | | 58, -44, 38 | | 4.85 | .054 | 856 | 4.55e-05 |
| ***Cortex*** | | 60, -56, 38 | | 4.62 | .114 |  |  |
|  |  | 58, -52, 20 | | 4.15 | .417 |  |  |
|  | | -40, -70, 42 | | 4.11 | .453 | 319 | .014 |
|  |  | -40, -62, 26 | | 4.02 | .543 |  |  |
|  |  | -44, -56, 30 | | 4.01 | .561 |  |  |
| ***posterior*** | | 12, -44, 30 | | 5.48 | .004 | 1564 | 1.24e-07 |
| ***cingulate cortex*** | | -4, -42, 36 | | 4.51 | .161 |  |  |
|  | | 8, -44, 30 | | 4.51 | .161 |  |  |

**Figure S1 Observed choice and simulated data based on inferred parameters.** Upper and lower left panels display data of high- and low-impulsive groups both showing aspects of model-free and model-based control in first-stage stay-switch behavior. Upper and lower right panels show simulated data based on inferred parameters of the hybrid model demonstrating that the hybrid model nicely recovers the actually observed behavior.

References

1. Daw ND, Gershman SJ, Seymour B, Dayan P, Dolan RJ. Model-based influences on humans' choices and striatal prediction errors. *Neuron* 2011; **69**(6)**:** 1204-1215.

2. Voon V, Derbyshire K, Ruck C, Irvine MA, Worbe Y, Enander J*, et al*. Disorders of compulsivity: a common bias towards learning habits. *Mol Psychiatry* 2014.

3. Wunderlich K, Smittenaar P, Dolan RJ. Dopamine enhances model-based over model-free choice behavior. *Neuron* 2012; **75**(3)**:** 418-424.

4. Deserno L, Huys Q, Boehme R, Buchert R, Heinze HJ, Grace AA*, et al*. Ventral striatal presynaptic dopamine reflects behavioral and neural signatures of model-based control during sequential decision-making. *Proc Natl Acad Sci U S A* 2015.

5. Sutton RS, Barto AG. *Reinforcement Learning: An Introduction*. MIT Press: Cambridge, MA, 1998.

6. Huys QJ, Cools R, Golzer M, Friedel E, Heinz A, Dolan RJ*, et al*. Disentangling the roles of approach, activation and valence in instrumental and pavlovian responding. *PLoS Comput Biol* 2011; **7**(4)**:** e1002028.

7. Huys QJ, Eshel N, O'Nions E, Sheridan L, Dayan P, Roiser JP. Bonsai trees in your head: how the pavlovian system sculpts goal-directed choices by pruning decision trees. *PLoS Comput Biol* 2012; **8**(3)**:** e1002410.

8. Radenbach C, Reiter AMF, Engert V, Sjoerds Z, Villringer A, Heinze HJ*, et al*. The interaction of acute and chronic stress impairs model-based behavioral control. *Psychoneuroendocrinology* 2015.

9. Stephan KE, Penny WD, Daunizeau J, Moran RJ, Friston KJ. Bayesian model selection for group studies. *Neuroimage* 2009; **46**(4)**:** 1004-1017.
